# Measuring Iron Oxide Composites with a Custom-Made Scanning Magnetic Microscope

**DOI:** 10.3390/s25082594

**Published:** 2025-04-19

**Authors:** Christian D. Medina, Leonardo A. F. Mendoza, Cleânio Luz-Lima, Antonio C. Bruno, Jefferson F. D. F. Araujo

**Affiliations:** 1 Department of Physics, Pontificia Universidade Católica do Rio de Janeiro, Rio de Janeiro 22451-900, RJ, Brazil; christiandavidmedina1993@gmail.com (C.D.M.); acobruno@gmail.com (A.C.B.); 2 Department of Physics, School of Physics, Faculty of Sciences, Central University of Venezuela—U.C.V., Caracas 1041, Venezuela; 3 Department of Electrical Engineering, State University of Rio de Janeiro—UERJ, Rio de Janeiro 20550-900, RJ, Brazil; mendonza@ele.puc-rio.br; 4 Physics Department, Federal University of Piauí, Teresina 64049-550, PI, Brazil; cleanio@ufpi.edu.br

**Keywords:** scanning magnetic microscope, Hall sensor, magnetic measurements

## Abstract

We improved a scanning magnetic microscope built previously by adding a new detection system and the capability of mapping samples applying magnetic fields from −500 mT to +500 mT. The mechanical structure was also enhanced to decrease vibrations of the system in the earth’s magnetic field. The microscope is based on a differential arrangement of two Hall effect elements. The overall system presented a sensitivity of about 850 $nT_{rms}\surd Hz$, and it was calibrated using a 99% pure nickel sphere. The system achieved a magnetic moment sensitivity of the order of 10 nAm^2^. All equipment used for operating the magnetic microscope was controlled by using the LabVIEW^®^ platform. We also fabricated samples with controlled properties using iron oxide microparticles and epoxy resin with various densities. We obtained the magnetization curves of the composites using the assembled microscope and compared them with the iron oxide powder.

## 1. Introduction

Scanning magnetic microscopes (SMMs) are used to obtain magnetic properties of materials. These properties have applications in different areas such as medicine [1], biophysics [2], materials science [3], and geology [4,5], among others. Older techniques still play an important role in obtaining these properties [6,7,8,9,10]. Among the instruments that are commercially available, the choice depends on a series of factors such as the required sensitivity, the mass and size of the sample, the temperature, the available budget, and the measurement speed. Instruments based on a vibrating sample magnetometer (VSM), the Superconducting Quantum Interference Device (SQUID), the Hall effect, the Kerr effect, a search coil, and alternating gradient are among the most used in commercial magnetometers [6,11,12,13,14].

The main difference between SMMs and magnetometers is that magnetometers measure the total magnetic moment of the whole sample and SMMs measure a detailed magnetic map in real time over the sample surface, usually with a spatial resolution of a few hundred microns. From magnetic maps, we can also obtain the essential properties that govern the magnetic behavior of materials, such as remanent magnetization, saturation magnetization, and coercive fields, including magnetic anisotropies. The extra time required to measuring magnetic maps provide us with an increased spatial resolution of the magnetic properties of the sample [4,5,8,9,10].

We demonstrate in the present work that it is possible to build a magnetic microscope, with a satisfactory performance, based on Hall effect elements using custom-made electronics along with a few other types of equipment that are usually present in a research laboratory. We assembled and calibrated an SMM consisting of an adjustable gradiometer system, improved readout electronics, two linear position stages, and an electromagnet that can apply magnetic fields from −500 mT to +500 mT while scanning the sample. In addition, to have samples with controlled parameters, we fabricated cubes consisting of a mineral powder embedded in a nonmagnetic medium.

## 2. Experimental Methods

Our SMM works based on two low-cost Hall effect sensors [4,5] wired in an axial gradiometric configuration. It has customized electronic circuits for biasing the sensors and detection of the induced field.

### 2.1. Structure of the Microscope

**Figure 1:**
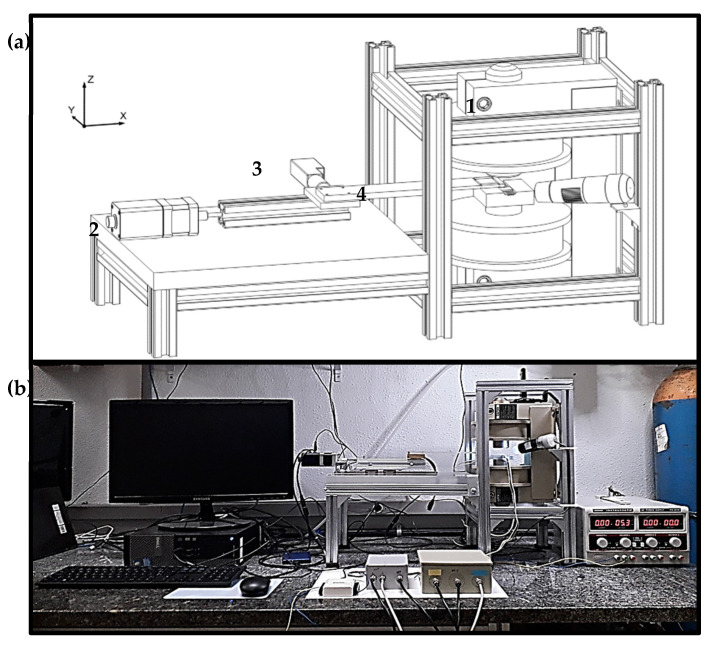
(**a**) In the figure, a solid aluminum frame is schematically shown, which holds the electromagnet (1) and a thick acrylic slab that supports the X-Y scanning mechanism (2–3); the sample holder is connected to the scanning mechanisms through a long acrylic arm (4). (**b**) Photograph of the scanning magnetic microscope.

The Hall SMM shown schematically in Figure 1a is housed inside a solid aluminum frame, which holds the electromagnet (1) and a thick acrylic slab that supports the X-Y scanning mechanism (2–3). The Hall sensors are attached to the lower electromagnetic pole, and the sample holder is connected to the scanning mechanisms through a long acrylic arm (4). The whole system sits on a 25 mm thick marble tabletop, as shown in Figure 1b.

We used a small electromagnet (GMW (San Francisco, CA, USA), model 3470). The current source used to drive it was an Agilent model E3634A (Santa Clara, CA, USA). For currents greater the 3.5 A, water cooling is needed, so we used the refrigerator QUIMIS model Q214S2 (Diadema, Brazil). When driving the electromagnet with the maximum current of 5.0 A, it generates a magnetic field of ±500 mT between the poles placed 7.0 mm apart. To measure the magnetic maps, our system stops at each position and acquires 5 samples, then continues for the next position of the grid. The sample holder is displaced using two Zaber linear actuators (Vancouver, BC, Canada) (models X-LSM100A-S and T-NA08A50 for the *X*-axis and *Y*-axis, respectively). One of the Hall elements is placed just over the sample, and the other is placed about 2.0 mm from the first one. The two signals are subtracted, eliminating the field due to the electromagnet and leaving just the induced field generated by the sample. Custom readout electronics [5] biased the Hall elements and detected the induced field. A LabVIEW^®^ program was developed to automate the equipment.

### 2.2. Readout Electronic System

**Figure 2:**
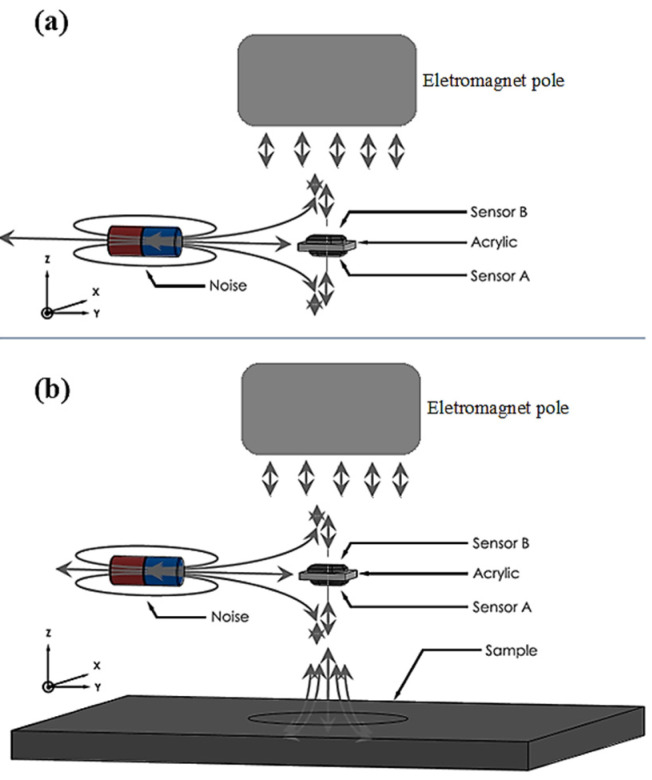
(**a**) Image illustrating the magnetic field lines of the electromagnet, the external noise lines, and the gradiometer system composed of two sensors with no sample. (**b**) Image representing the magnetic field lines in the presence of the sample. In both figures, the reader should assume that the magnetic field applied by the electromagnet must be uniform.

The Hall effect elements were AC biased at 1.0 kHz, and the induced field was phase-sensitive detected. The biasing, modulation/demodulation, and filtering electronics [5] used the AD620 to amplify the input signal: the AD630 demodulates it and the OP27 instrumentation amplifier is responsible for producing the DC output signal and filtering [5]. The system gain can be adjusted, and it is powered with a commercial DC voltage source. In our experience, our custom circuit is a cost-effective alternative with satisfactory results, as shown in this work. A circuit board for performing the Hall effect gradiometer was also used, as is shown schematically in see Figure 2. The role of the Hall effect gradiometer is to eliminate the electromagnet’s field and ambient magnetic noise, which are uniform in space, so for these sources, sensor A minus sensor B reads nearly zero, as is illustrated in Figure 2a, and both sensors have a spatial resolution of 300 µm [4]; however, when the sample is positioned close to sensor A, only this sensor detects the sample-induced field, as shown in Figure 2b. In both figures, the reader should assume that the magnetic field applied by the electromagnet must be uniform. The last element of the system is the data acquisition board, National Instruments USB-6210 (Austin, TX, USA), that collects the scanning positions, the Hall gradiometer output, and the signal of a third Hall sensor (Melexis MLX 90251 (Ypres, Belgium)) dedicated to measuring the applied field by the electromagnet.

### 2.3. Calibration

Sample characterization was performed by obtaining the magnetic moment as a function of the applied magnetic field; however, an appropriate theoretical model with the sample geometry was necessary to obtain this quantity, and in addition, it is important to know beforehand the distance between the sample and the sensor so that the only variable in the theoretical model is the magnetic moment [4,5,15,16,17]. The vertical distance from the sample to the sensor is an unknown value in our system because the sensitive element of the sensor (sensor A) is encapsulated inside the Hall chip. Although the distance between the sensor surface and the sample is known, we are not sure of the spacing between the encapsulation surface and the Hall element. Therefore, before making measurements with the microscope, it was necessary to calibrate the device by using a sample with known magnetic moment in order to find this distance. The known source is a 99% pure nickel sphere with a 3 mm diameter, whose magnetic moment is available in the literature [NIST Standard Reference Material^®^ 772a]. Using the measurements and a theoretical model appropriate to the geometry of the sphere, we calibrated the microscope and found the unknown distance. The magnetic dipole model was the theoretical model chosen, located in the center of the sphere [17,18]. The magnetic dipole located at the origin of the coordinate system is at a distance ***r (x, y, z)*** = x ***i*** + y***j***+ z ***k*** and has a magnetic moment $m_{z}$ that points in the direction of the positive *z*-axis.

The magnetic field can be determined using the following equation [17]:

(1) $$B(r)=\frac{\mu_{0}}{4\pi }[3(m\;\cdot \;r)r\frac{1}{r^{5}}-m\;\frac{1}{r^{3}}]$$

where $\mu_{0}$ is the permeability in vacuum, and we should remember that the magnetic moment is in the direction of the *z*-axis **m** = $m_{z}$***k***. Thus, Equation (1) can be rewritten as follows:

(2) $$B_{z}\left(x,\;y,\;z\right)=\frac{\mu_{0}}{4\pi }[3\frac{m_{z}\left(z-z_{0}\right)z_{0}}{{\left[{\left(x\right)}^{2}+{\left(y\right)}^{2}+{\left(z\right)}^{2}\right]}^{5/2}}-\frac{m_{z}}{[{\left(x\right)}^{2}+{\left(y\right)}^{2}+{\left(z{)}^{2}\right]}^{3/2}}]$$

**Figure 3:**
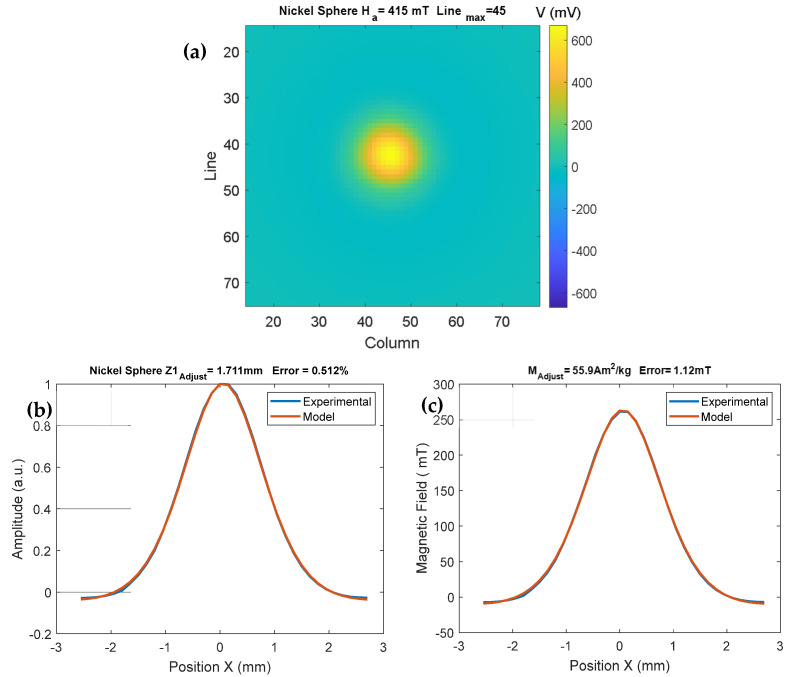
(**a**) Magnetic map of the nickel sphere applying a field of 415 mT. We can observe that line 45 was the maximum intensity line of the induced magnetic field. (**b**) In this graph, we can observe that the distance was 1.711 mm, with an error of 0.512%. (**c**) The graph shows the field for a sphere magnetization of 55.9 Am^2^/kg.

We also calculated the magnetic flux that crosses the sensitive area of the Hall sensor.

(3) $$d\Phi_{z}\left(x,\;y,\;z\right)=\frac{\mu_{0}}{4\pi }\;[3\frac{m_{z}\left(z-z_{0}\right)z_{0}}{{\left[{\left(x\right)}^{2}+{\left(y\right)}^{2}+{\left(z\right)}^{2}\right]}^{5/2}\;}-\frac{m_{z}}{[{\left(x\right)}^{2}+{\left(y\right)}^{2}+{\left(z{)}^{2}\right]}^{3/2}}]dxdy$$

where the coordinates (x, y, z) represent the position of the sensor center measured from the dipole position. Through Equations (2) and (3), we created a subroutine written on the MatLab^®^ platform (R2023b). This subroutine can simulate the magnetic dipole curve when it is magnetized by the electromagnet and displaced by the linear actuators (see Figure 3). In the first phase of the subroutine, a normalization of Equation (2) was performed. In this way, we could find the distance between the sensor and the sample; then, with this information, the modeling process was performed, and we found the magnetic moment using Equation (3).

Figure 3a shows the magnetic map experiment of the 99% pure nickel sphere, which was attached to a 26 mm square sample holder; this sample holder was connected to an acrylic arm attached to the scanning mechanism (see Figure 1a). In the center of this sample holder, a cylindrical cavity of 3 mm in diameter by 3 mm in thickness was constructed where the nickel sphere was fixed. This map was obtained using a step of 150 µm in both axes. The program found the position of the maximum intensity line of the induced magnetic field of the measurement. The distance between the sensor and the sample depends only on geometrical parameters, so it can be obtained through normalization of the signal (see Figure 3b). An error of 0.52% was found between the experimental and model signals using a least-squares fitting routine. Afterwards, to confirm the obtained distance value, we denormalized the signal and found, through a least-squares fitting, a magnetization of 55.9 Am^2^/kg for the sphere at a field of approximately 415 mT (Figure 3c). The NIST magnetization standard for an applied magnetic field of 500 mT is 54.9 Am^2^/kg for a 99.99% pure nickel sphere [NIST Standard Reference Material^®^ 772a]. Since the nickel sphere is already in the saturation region at 415 mT, the error in this process is approximately 1.78%.

**Figure 4:**
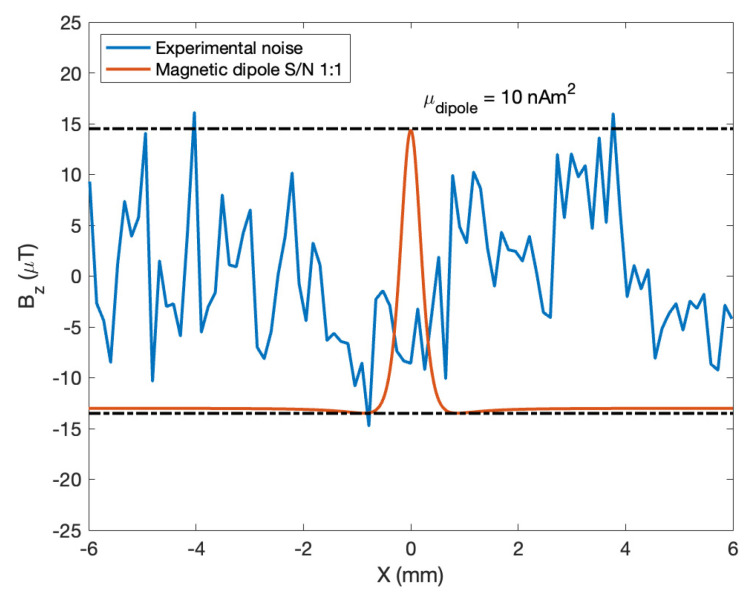
Shows the actual system noise and the equivalent magnetic dipole we used to estimate the system sensitivity using the 1:1 signal-to-ratio criteria. The sensitivity value estimated was 11 nAm^2^.

We also estimated the overall microscope sensitivity in terms of a magnetic dipole moment using the 1:1 signal-to-ratio criteria. The result can be seen in Figure 4.

## 3. Synthesis of Iron Oxide Composites

To simulate a rock sample, we fabricated a composite in a cubic shape containing iron oxide microparticles mixed with epoxy with different weight ratios, which will be mapped and characterized. To produce the iron oxide composites, we used the following materials: iron oxide microparticles (RW222, MetalChek (Bragança Paulista, Brazil)), a Teflon mold in a cubic shape, adhesive tape, epoxy (hardener and resin), and an electronic scale (FX-40, A&D). The general procedure for creating the composite cubes is described as follows: the Teflon mold is prepared by placing adhesive tape on the edges of the opening to facilitate the extraction of the cube at the end of the procedure. The base and mold weights are individually measured. Resin and hardener are placed in the base in equal quantities (one drop of each). The weight of the base with the content is measured again. The iron oxide microparticles are placed inside the base but away from the glue, and the weight is measured once again. The base content is mixed, using a small spatula, for approximately 1 min, until obtaining a very homogeneous texture. Subsequently, the mixture is placed inside the Teflon mold. When the mold cavity is full, the mold and the base weights are measured with the residues separated. Finaly, the cube inside the mold should rest for about 5 days.

**Figure 5:**
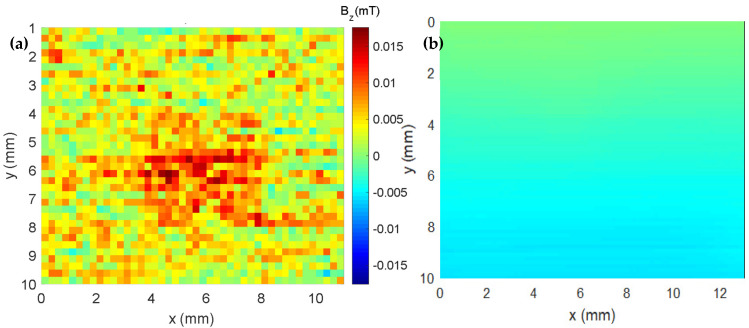
(**a**) Cube map made only with epoxy. (**b**) Magnetic map without any samples.

Using the above-described method, samples with different proportions of epoxy and iron oxide microparticles were made. In addition, a cube of the epoxy resin with the same dimensions was fabricated. The remanent magnetization of the epoxy cube was under 15 µT, as can be seen in Figure 5a. However, this does not mean that the epoxy will have such low induced fields in the presence of high applied magnetic fields. We assumed the mixture of epoxy resin and microparticles was homogeneous during production and calculated their proportions accordingly. A magnetic map without a sample was measured (see Figure 5b) to show the uniformity of the field without the presence of a sample.

**Figure 6:**
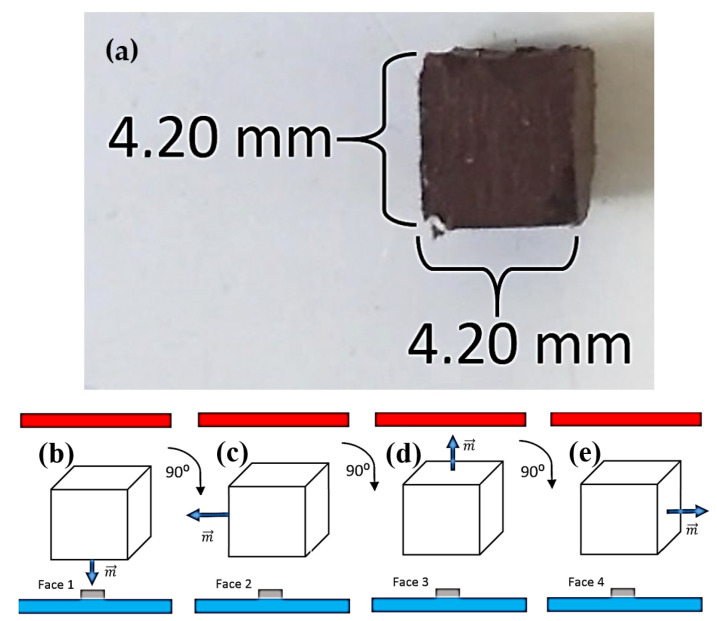
(**a**) Photograph of the iron oxide composites produced in the PUC-Rio. (**b**) Schematic of the mapping of the iron oxide composites showing the rotation of the sample in each measurement, with the north and south pole of the electromagnet in red and blue, respectively, the Hall A sensor of the gradiometer in gray color, and the direction of magnetization. Mapping of face 1; (**c**) mapping of face 2; (**d**) mapping of face 3; (**e**) mapping of face 4.

Each composite was mapped using the Hall microscope. The measurement was made as follows: Before mapping, the cube was fixed on the acrylic sample holder and placed inside the electromagnet. For this first test, we used a composite with approximately 83.5 mg of mass; the epoxy resin mass corresponds to approximately 55.4 mg of the total mass. Thus, we assume that the amount of mass of microparticles in this cube is approximately 28.1 mg. For this composite, we measured the length of its edges and obtained a cube with approximately the following dimensions: 4.20 mm × 4.20 mm × 4.20 mm (see Figure 6a).

The sample was magnetized with an external field of approximately 500 mT, which is the maximum magnetic field produced by our electromagnet. This was carried out to magnetize the sample until close to saturation. After magnetizing the cube, the electromagnet was turned off, and the sample was left with a remanent field. The cube was then mapped face to face closer to the sensor, which we call ‘face 1’ (Figure 6b).

After obtaining the first map, the position of the cube was changed by rotating 90 degrees, and, maintaining the same conditions as the previous measurement, face 2 was mapped (Figure 6c). Repeating the cube rotation procedure, we mapped face 3 (Figure 6d) and face 4 (Figure 6e) of the cube, obtaining a total of four magnetic maps per cube, where the gradiometer reads the remanent magnetic field of the composite.

**Figure 7:**
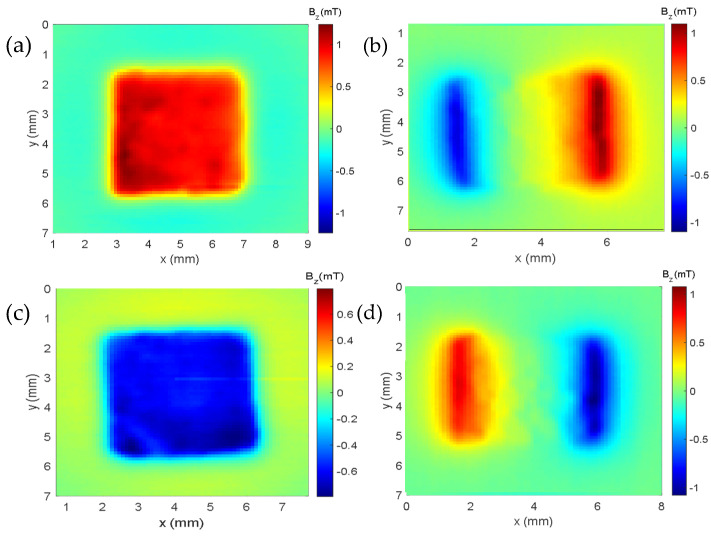
Magnetic map of the sample (28.1 mg of iron oxide microparticles): (**a**) map of face 1; (**b**) map of face 2; (**c**) map of face 3; (**d**) map of face 4.

In the cube map with only epoxy resin, it was subjected to an external field of 500 mT. After turning off this 500 mT field, the epoxy cube was magnetically mapped, and a small remanence field of approximately 15 µT was detected. From all the samples analyzed, we chose an intermediate (sample 3, see Table 1) composite to be characterized by our Hall microscope. In Figure 7a–d, we can observe the magnetic maps of faces 1, 2, 3, and 4, respectively. The intensity of the remanent magnetic field of the sample was around 1.2 mT for face 1. To obtain the magnetic moment of the cube containing 28.1 mg of magnetic microparticles, we followed the same procedure performed for calibrating the device. This procedure of measuring the remanent field can be useful in various experiments involving steel and rock samples [4,5,19,20].

**Figure 8:**
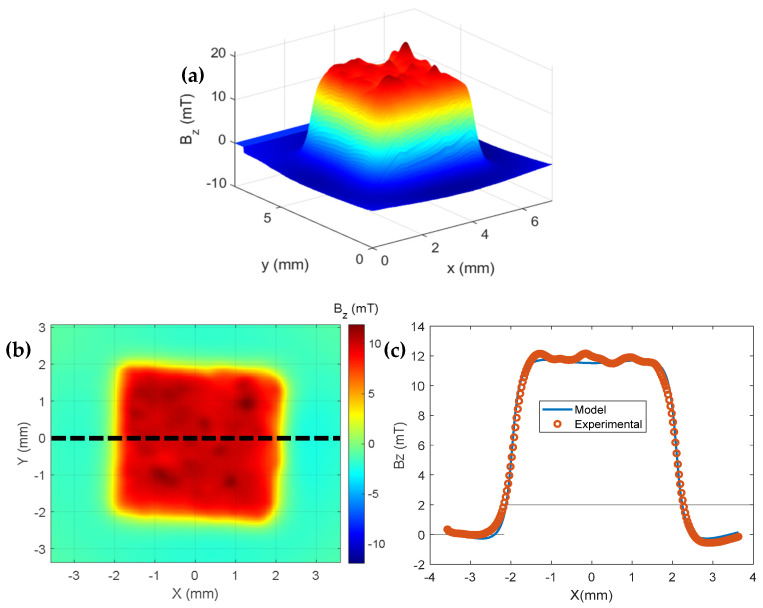
(**a**) Magnetic map in 3 dimensions showing the small peaks formed at the top of the image. (**b**) Magnetic map of the sample with a horizontal line crossing the region of medium iron oxide composites. (**c**) Graphs of the magnetic field as a function of the position of the *X*-axis displacement of the sample obtained with the Hall microscope (blue) and the rectangular prism theoretical model (red).

We placed the cube with the composite in the sample holder again to map it using the Hall microscope, applying (Figure 8a) external fields from 500 mT to −500 mT and back to 500 mT. We scanned the cube using a step of 140 µm in the linear actuators in the X and Y directions. After obtaining the map, a grid was made to determine the central region of the sample, and we used just this line to compare the experimental data to our model (see Figure 8b).

Previously, we mentioned that the characterization of a sample, in terms of its magnetization curve, basically depends on obtaining a magnetic moment as a function of the applied field; however, in our research, we used the magnetic moment of a cube-shaped model (Figure 8c) uniformly magnetized on the vertical axis [17]. On the MATLAB^®^ platform, the component $B_{z}$(x, y, z) of the magnetic field, produced by the cube-shaped model with uniform magnetization $M_{z}$, was written using Equation (4) [17]:

(4) $$B_{z}(x,\;y,\;z)=-\frac{\mu_{0}M_{z}}{4\pi }\;[F(-x,\;y,\;z)\;+\;F(-x,\;y,\;-z)]+\;F(-x,\;-y,\;z)\;\;+\;F(-x,\;-y,\;-z)+\;F(x,\;y,\;z)\;+\;F(x,\;y,\;-z)\;F(x,\;-y,\;z)\;+\;F(x,\;-y,\;-z)]$$

The function F is defined using the following equation [17]:

(5) $$F(x,\;y,\;z)=\arctan\frac{\left(x+a\right)\left(y+b\right)}{\left(z+c\right)\sqrt{{\left(x+a\right)}^{2}+{\left(y+b\right)}^{2}+{\left(z+c\right)}^{2}}}$$

**Figure 9:**
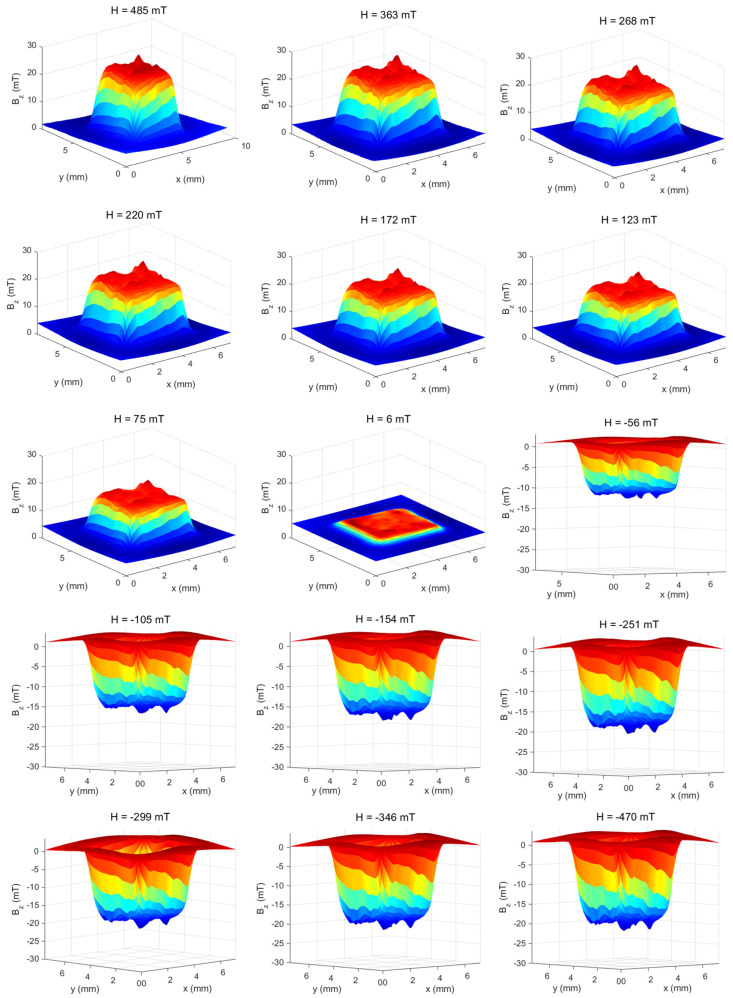
Magnetic maps of the cube-shaped sample with the magnetic field ranging from 485 mT to −470 mT.

In Equation (5), the terms *a*, *b*, and *c* represent half of the sides of the cube. Using Equations (4) and (5), a subroutine was designed, which was used for adjustment to obtain the magnetic moment of the rectangular-shaped sample [17]. After adjusting the theoretical model with the experimental model (Figure 7c), we used the model described above to obtain the magnetic moment of the sample, making the magnetic maps of face 1. Approximately 37 magnetic maps were made with a field between 485 mT and −470 mT. For each map, the magnetic moments were obtained using the model of a prism with a rectangular base. We can observe the change in the intensity of the induced magnetic field in Figure 9, which represents the magnetic maps of the cube-shaped sample, with the magnetic field between 485 mT and −470 mT.

**Figure 10:**
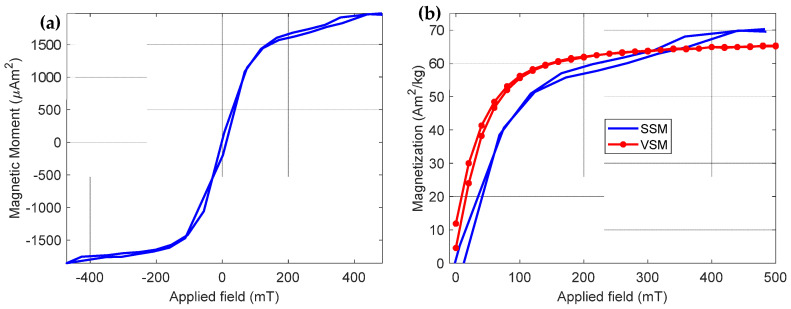
(**a**) Curve representing the magnetic moment of the cube regarding the magnetic field applied by the scanning magnetic microscope. (**b**) Graphs representing the magnetization of the results obtained from the magnetic moments divided by the mass of iron oxide (continuous curve in blue color) and the magnetization curve of the iron oxide powder sample, measured in the VSM, model 7404 (continuous and dotted curve in red color).

After obtaining the magnetic moments, it was possible to construct a curve of the magnetic moment versus the applied magnetic field (see Figure 10a). In this curve, it is possible to verify that there is a symmetry between the values of magnetic moments (positive) for positive magnetic fields and the values of negative magnetic moments for negative magnetic fields; this symmetry behavior is a standard behavior among magnetization curves. We can observe in Figure 10b that the magnetization curve of the iron oxide composites is different from the curve of the microparticles, and the magnetic moment of the iron oxide composites is approximately 69.8 Am^2^/kg for an applied field of 441 mT, a remanence of approximately 1.0 Am^2^/kg, and a coercive field of 12 mT. It is worth mentioning that this is the curve of the iron oxide composites (iron oxide microparticles + epoxy). For comparison purposes, we present the graph in Figure 10b, which shows the magnetization curve of only the iron oxide microparticles that were measured in the vibrating sample magnetometer—VSM (Lakeshore Inc., Gaffney, SC, USA, model 7404).

**Figure 11:**
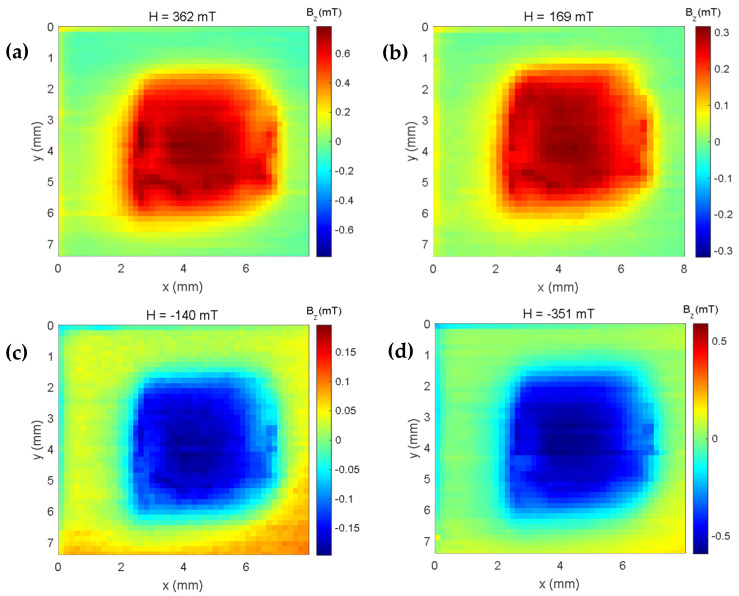
Magnetic maps of the induced field when (**a**) a positive field of 362 mT, (**b**) a positive field of 169 mT, (**c**) a negative field of −140 mT, and (**d**) a negative field of −351 mT are applied to the epoxy sample.

This difference in the magnetization curve of the iron oxide composites and the iron oxide microparticles may be related to the contribution of the epoxy material; even if it has a low remanence magnetization (see Figure 5), the same does not happen for more intense fields, as can be seen in the maps in Figure 11, where we can verify the intensity of the induced magnetic field of the cube sample containing only epoxy.

## 4. Conclusions

We assembled a scanning magnetic microscope that uses two Hall sensors configured as an axial gradiometer. The system is fully controlled using programs developed in LabVIEW^®^. The mechanical structure was improved from an earlier design. The readout electronics were improved using three custom-made three printed circuit boards, turning our microscope independent of other commercial instruments. We found a distance of a few hundreds of micrometers between the GaAs sensor element of sensor A and its surface using a 99% pure nickel sphere. The magnetic moment sensitivity of our Hall microscope was estimated to be about 10 nAm^2^. In addition, we found a system white noise of 850 nT_rms_/(Hz)^½^. We manufactured cubes composed of iron oxide microparticles and epoxy to simulate a rock sample. The cube-shaped samples were modeled assuming a homogeneous iron oxide powder distribution. We obtained about 70 Am^2^/kg for the magnetization saturation of the cube at an applied field of 441 mT.

## Figures and Tables

**Table 1:** Specifications of iron oxide composites made at PUC-Rio.

Sample	Microparticles + Epoxy Cube Mass (mg)	Microparticle Mass	Epoxy Mass (mg)
0	70.0	0	70
1	95.9	38.1 mg	57.8
2	90.7	31.4 mg	59.3
3	83.5	28.1 mg	55.4
4	86.5	8.83 mg	77.7
5	81.9	2.36 mg	79.5
6	72.3	695 µg	71.6

## Data Availability

Data are contained within the article.
